# Supplementary material for: Integrated Single-Cell Transcriptome Analysis Reveals Novel Insights into the Role of Opioid Signaling in the Pathophysiology of Inflammatory Bowel Disease
Source: Biomedicines. 2025 Jun 6;13(6):1398. doi: 10.3390/biomedicines13061398 (PMC12190152; doi:10.3390/biomedicines13061398)
Supplement: Supplementary file 1 [file biomedicines-13-01398-s001.zip › Supplementary Figures.pdf]

Supplementary Figure

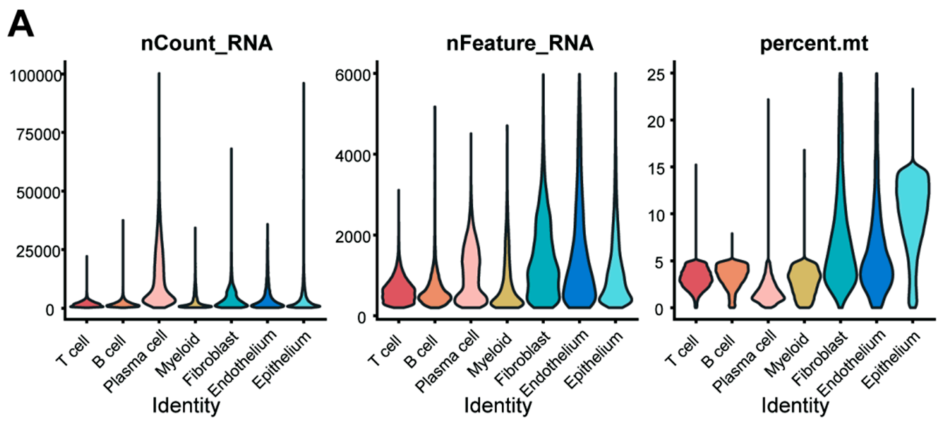

Figure S1. Violin plot of all cell types after quality control. (A) Violin plot of RNA count of all cell types; (B) Violin plot of RNA feature of all cell types; (C) Violin plot of mt gene percentage of all cell types.

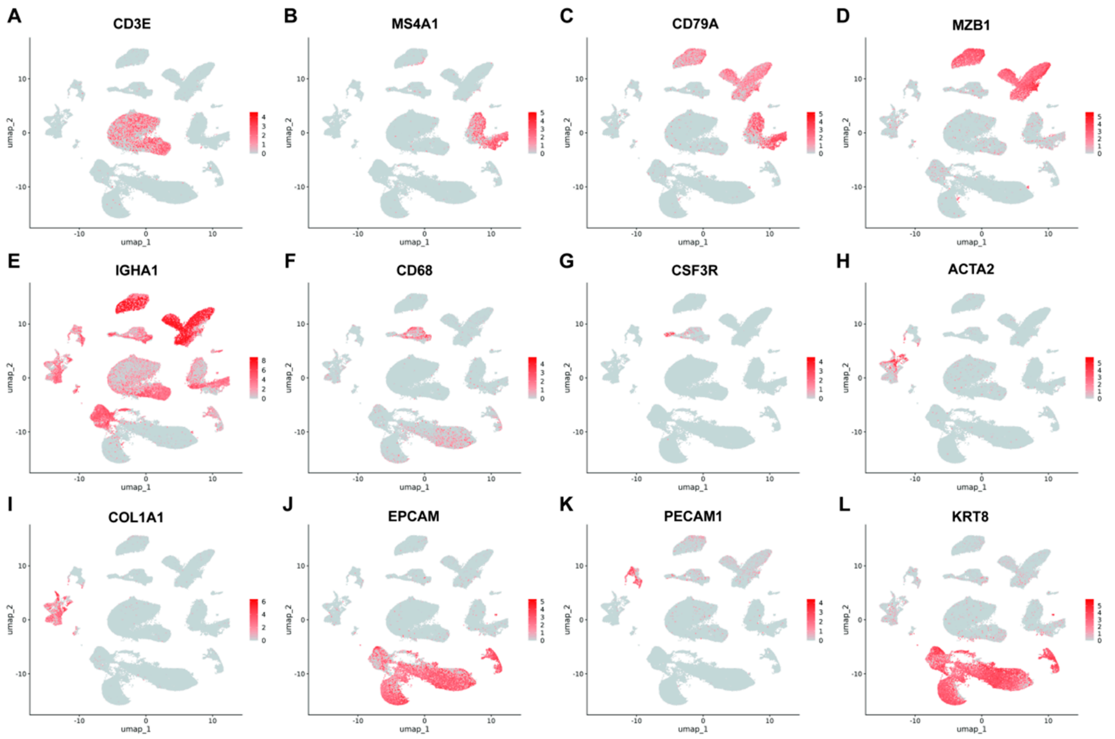

Figure S2. The UMAP of different cell markers displayed distribution of the cells among CD, UC, and NC. (A) The UMAP of CD3E displayed distribution of T cells; (B-C) The UMAP of MS4A1 and CD79A displayed distribution of B cells; (D-E) The UMAP of MZB1 and IGHA1 displayed distribution of plasma cells; (F-G) The UMAP of CD68 and CSF3R displayed distribution of myeloid cells; (H-I) The UMAP of ACTA2 and COL1A1 displayed distribution of fibroblast cells; (J) The UMAP of PECAM1 displayed distribution of endothelium cells; (K-L)The UMAP of EPCAM and KRT8 displayed distribution of epithelium cells. UMAP plots visualize high-dimensional transcriptomic data in 2D space, where proximity indicates transcriptional similarity. Cluster labels were assigned based on canonical marker genes and colors correspond to cell subgroups where the marker genes are located.

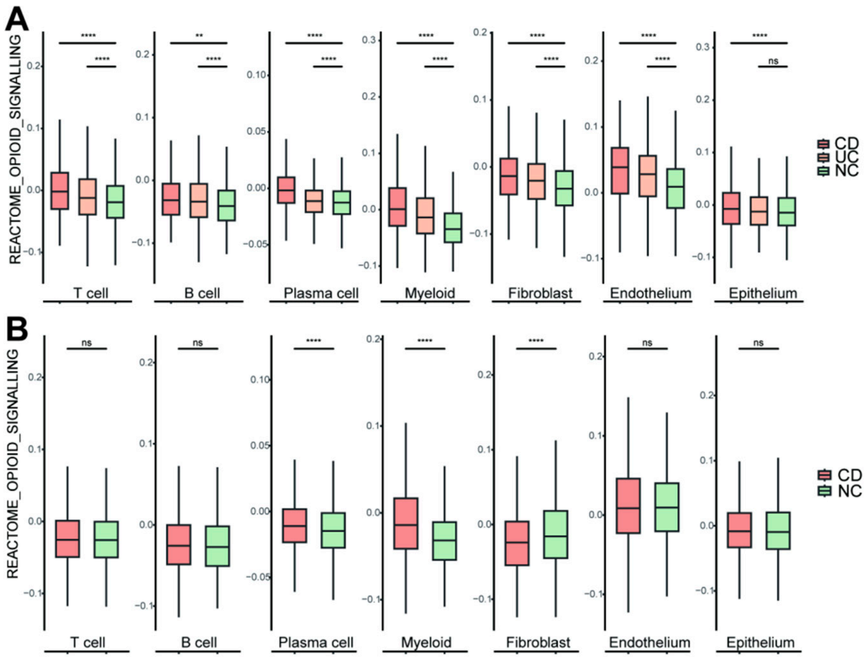

Figure S3. Opioid signalling-related genes were significantly upregulated in myeloid cells in IBD. (A) The opioid signalling scores of each cell group across NC, UC and CD in colorectum; (B) The opioid signalling scores of each cell group across CD and NC in ileum.

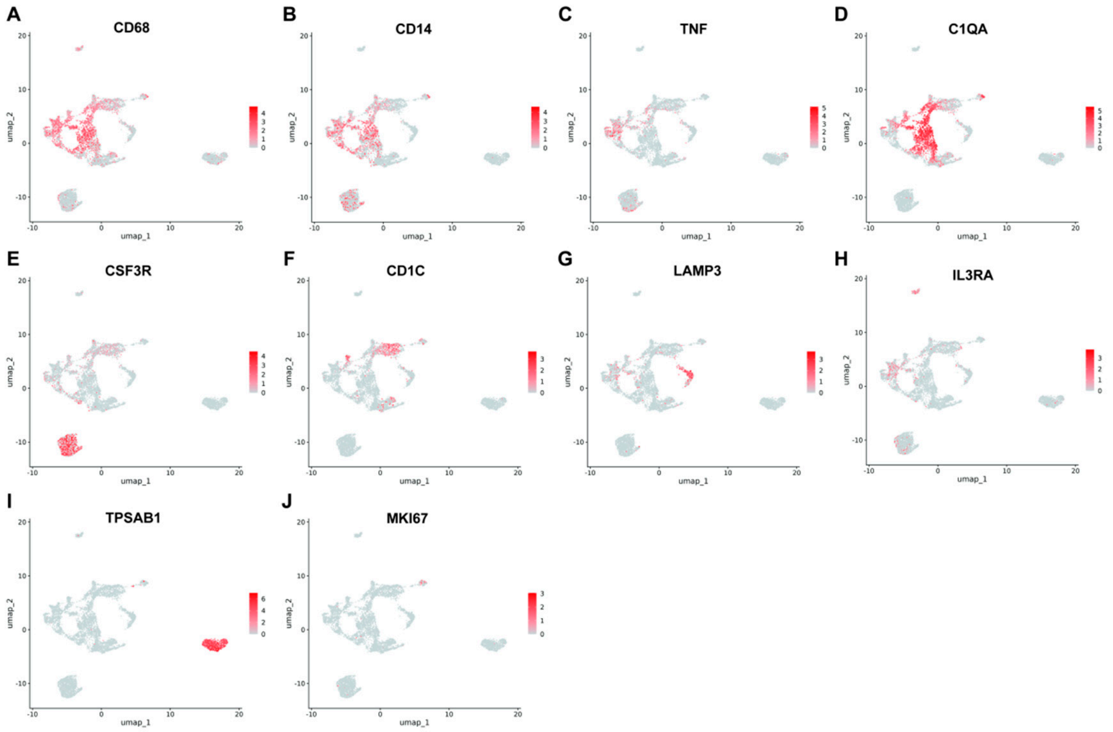

Figure S4. The expression of marker genes used to define myeloid cell subgroups. (A-D) The UMAP of CD68, CD14, TNF, C1Q displayed distribution of monocytes and macrophages; (E) The UMAP of CSF3R displayed distribution of neutrophils; (F) The UMAP of CD1C displayed distribution of cDC2 cells; (G) The UMAP of LAMP3 displayed distribution of cDC3 cells; (H) The UMAP of IL3RA displayed distribution of pDC cells; (I) The UMAP of TSPAB1 displayed distribution of Mast cells; (J) The UMAP of MKI67 displayed distribution of cycling myeloid cells. UMAP plots visualize high-dimensional transcriptomic data in 2D space, where proximity indicates transcriptional similarity. Cluster labels were assigned based on canonical marker genes and colors correspond to cell subgroups where the marker genes are located.

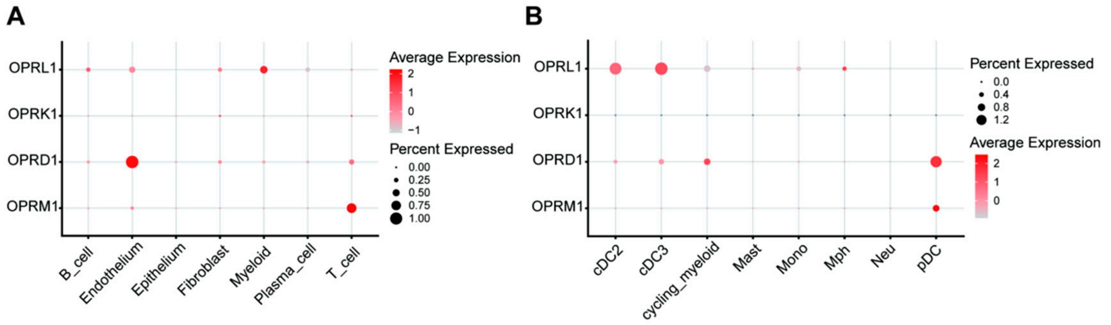

Figure S5. The expression patterns of opioid receptor subtypes across diverse cell types. (A) Analysis of the expression profiles of opioid receptor subtypes in diverse cell types showed that MOR was specifically expressed in T cells, ORL-1 exhibited high expression in myeloid cells, and DOR showed high expression levels in endothelial cells; (B) Analysis of the expression profiles of opioid receptor subtypes in myeloid cells demonstrated that ORL-1 exhibited ubiquitous expression across all subpopulations of myeloid cells.

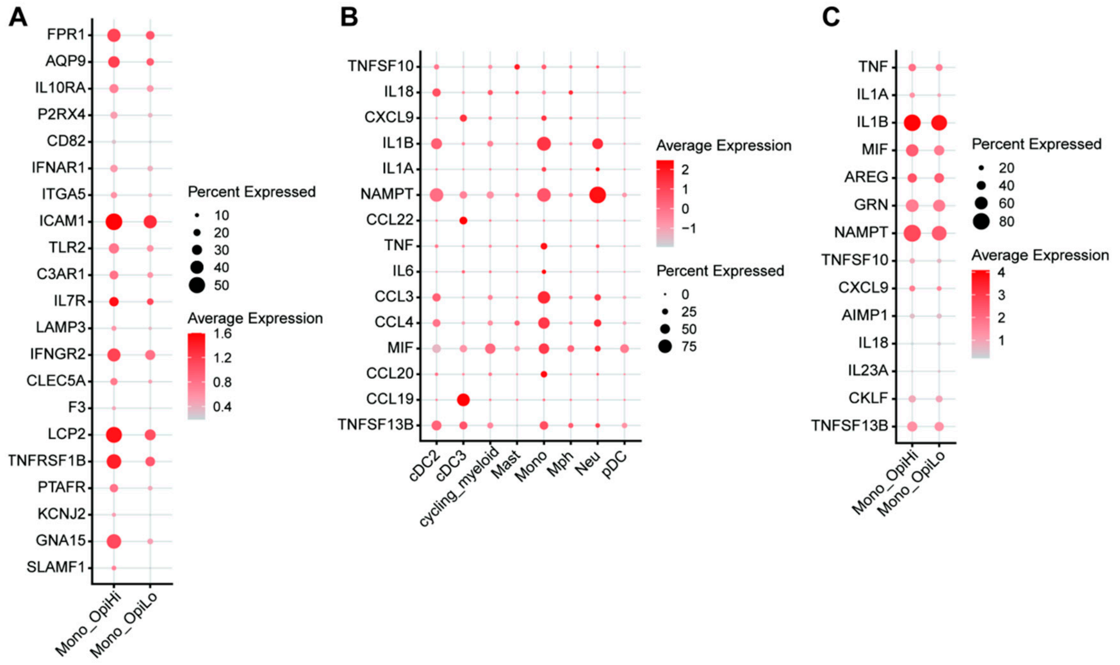

Figure S6. Analysis of the differences in cytokine and cell surface genes between OpiHi monocytes and OpiLo monocytes. (A) The analysis of cell membrane protein expression differences between OpiHi monocytes and OpiLo monocytes identified LCP2, TNFRSF1B, and GNA15 as promising candidates for cell surface marker detection in flow cytometry; (B) Analysis of the expression profiles of cytokine gene expression in myeloid cells demonstrated that Monocytes serve as the primary producers of IL-1β, TNF, and MIF cytokines within the myeloid cell population. (C) A further analysis of the differences in cytokine genes between OpiHi monocytes and OpiLo monocytes revealed that OpiHi monocytes are distinguished by their secretion of inflammatory factors (MIF, TNF, TNFSF10, and TNFSF13B).
